# The Regulation of Floral Colour Change in *Pleroma raddianum* (DC.) Gardner

**DOI:** 10.3390/molecules25204664

**Published:** 2020-10-13

**Authors:** Fernanda Mendes Rezende, Mads Hartvig Clausen, Magdalena Rossi, Cláudia Maria Furlan

**Affiliations:** 1 Department of Botany, Institute of Biosciences, University of São Paulo (USP), São Paulo 05508-060, Brazil; magdarossirosso@gmail.com; 2 Center for Nanomedicine and Theranostics, Department of Chemistry, Technical University of Denmark (DTU), 2800 Kgs. Lyngby, Denmark; mhc@kemi.dtu.dk

**Keywords:** Melastomataceae, anthocyanin, flavonol, chalcone synthase, flavonol synthase, anthocyanidin synthase, carbohydrates

## Abstract

Floral colour change is a widespread phenomenon in angiosperms, but poorly understood from the genetic and chemical point of view. This article investigates this phenomenon in *Pleroma raddianum*, a Brazilian endemic species whose flowers change from white to purple. To this end, flavonoid compounds and their biosynthetic gene expression were profiled. By using accurate techniques (Ultra Performance Liquid Chromatography-High-Resolution Mass Spectrometry (UPLC-HRMS)), thirty phenolic compounds were quantified. Five key genes of the flavonoid biosynthetic pathway were partially cloned, sequenced, and the mRNA levels were analysed (RT-qPCR) during flower development. Primary metabolism was also investigated by gas chromatography coupled to mass spectrometry (GC-EIMS), where carbohydrates and organic acids were identified. Collectively, the obtained results suggest that the flower colour change in *P. raddianum* is determined by petunidin and malvidin whose accumulation coincides with the transcriptional upregulation of early and late biosynthetic genes of the flavonoid pathway, mainly *CHS* and *ANS*, respectively. An alteration in sugars, organic acids and phenolic co-pigments is observed together with the colour change. Additionally, an increment in the content of Fe^3+^ ions in the petals, from the pink to purple stage, seemed to influence the saturation of the colour.

## 1. Introduction

**Figure 1:**
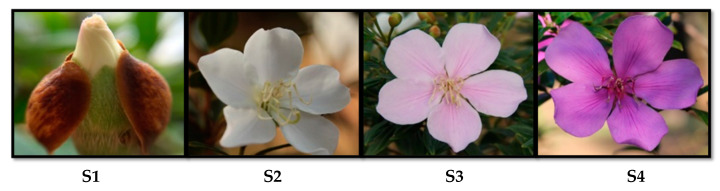
Stages of the floral colour change of *Pleroma raddianum*. S1—buds (day 0), S2—white flowers (day 1), S3—pink (day 2), S4—purple (day 3).

*Pleroma raddianum* (Melastomataceae, Myrtales), named as “manacá-da-serra”, is an endemic species from the Brazilian Atlantic Forest. The scientific name of this species, classically known as *Tibouchina pulchra* (Cham.) Cogn, has recently changed [1]. Its use for urban ornamentation is increasing due to its colourful flowers. In this sense, the ornamental dwarf cultivar of *P. raddianum* (“manacá-anão”) blooms with the same temporal pattern of floral colour as the wild parental: buds, the first stage (S1), open as white flowers (S2) that one day after become pink (S3), finally, from the third day towards senescence the flowers achieve purple colouration (S4) (Figure 1).

Previously, we characterised the phenolic pigments profile in *P. raddianum* flowers, which are mainly flavonoids (flavonols and anthocyanins), but also few phenolic acids. Thirty compounds were detected, there being twenty-three flavonoids identified by Ultra Performance Liquid Chromatography-High-Resolution Mass Spectrometry (UPLC-HRMS), eight of which were isolated and analysed by one- and two-dimensional nuclear magnetic resonance. Kaempferol derivatives were the major flavonols while petunidin *p*-coumaroylhexoside acetylpentoside and malvidin *p*-coumaroylhexoside acetylpentoside were the detected anthocyanins [2].

In general, the temporal alteration of flower colour is determined by the differential accumulation of anthocyanins and regulated by several factors, such as pollination [3], gene expression regulation [4,5,6,7], pH [8], presence of co-pigments and flavonoid-metal complexation [9].

Concerning pollinators, usually the colour change is associated with pollen viability [3]. In *P. raddianum* the pollen viability, stigma receptivity and fruit set were similar in white and pink flowers: large bees visited white flowers more frequently but at the end of the flowering season, when white flowers are scarce, the bees also visited pink flowers. This data suggest that flower colour change is not associated with pollination but visitor diversity enlargement in this species [10,11].

Among the species in which the corolla changes colour during flower development, the underlying molecular mechanism has only been explored for a few of them. In *Viola cornuta* L. (Violaceae) the flowers change from white to purple, and this change is related to the presence of pollen in the stigma, the incidence of light, and the differential expression of anthocyanin biosynthetic genes [12,13]. In *Nicotiana mutabilis* Stehmann and Semir (Solanaceae) flowers, the colour change from white to red, passing through different shades of pink, is determined by the upregulation of *CHALCONE SYNTHASE* (*CHS*) expression and the consequent anthocyanin accumulation [4]. Anthocyanin increment with the gain of pink, purple and red shades was described in other model plants [5,6]. Furthermore, other metabolites have shown to influence flower colour change, from white to pink, in *Paeonia ostii* T. Hong and J. X. Zhang (Paeoniaceae), such as alterations in carbohydrate and fatty acid contents, as well as ethylene signalling [5].

The colour change is not only related to pigments biosynthesis and accumulation, but also their catabolism, as reported for *Brunfelsia calycina* Benth. (Solanaceae) and *Malus hupehensis* (Pamp.) Rehder (Rosaceae) [8,14]. In *B. calycina*, the petals turn from dark purple to completely white within three days after anthesis, due to the reduction of anthocyanin concentration and increment of phenolic acid content during flower development [8,15]. A basic vacuolar peroxidase was identified as the putative responsible for the in planta degradation of anthocyanins [16].

The pH affects the final colour of flower petals by determining the conformation and absorption spectrum of anthocyanins. The light blue petals of morning glory (*Ipomoea tricolour* Cav., Convolvulaceae) owe their colour to the effect of the alkaline pH on the accumulated anthocyanins. The closed buds of these flowers are purplish–red and their cells have a pH of 6.6. However, when the flowers open, the petal cell pH increases up to 7.7, and the pigment changes colour to sky blue [17]. Recently, studying the genetic determinants of *Petunia hybrida* Vilm. (Solanaceae) flower pigmentation, Verweij et al. [18] have identified a WRKY-type transcriptional factor (PH3) that regulates the expression of the proteins responsible for the vacuole acidification.

Co-pigmentation can be defined as the formation of noncovalent complexes involving a pigment and a co-pigment, and the subsequent change in the optical properties of the pigment. Hydrolysable tannins, flavonoids, and phenolic acids are natural co-pigments. Flavonoids are the major class of co-pigments (flavonols, flavones, flavanols, and even dihydroflavonols) due to the extension of their π-conjugation over their entire tricyclic core structure (A, B, and C rings), being the most efficient flavones and flavonols (e.g., quercetin, kaempferol, isoquercitrin, and rutin) among them. Hydroxycinnamic acids and derivatives such as caffeic, *p*-coumaric, ferulic, sinapic, chlorogenic, and caffeic acids are commonly described as relatively efficient co-pigments as well. Some non-phenolic co-pigments have also been described, including alkaloids, amino acids, organic acids, nucleotides, and polysaccharides, but their efficiency is usually lower than polyphenols [9].

Some studies showed that metals can influence the colour by establishing a metalloanthocyanin complex. In cornflowers (*Centaurea* sp., Asteraceae), the bright blue colour is the result of a supramolecular structure with stacking between co-pigments, pigments and metals: six molecules of apigenin, six molecules of cyanidin and four metal ions (Fe^3+^, Mg^2+^, and two Ca^2+^) [19]. Hortensia flowers (*Hydrangea macrophylla* (Thumb.) Ser., Hydrangeaceae) is the best-known example of flavonoid–metal complexation influencing the corolla colour. Under high aluminium content and acidity of the soil, the flowers are blue due to a nonstoichiometric complex with Al^3+^, delphinidin 3-glucosides, and quinic acid derivatives, while upon low aluminium concentrations, the same metal-pigment-co-pigment complex became light pink, as the result of soil pH change [20,21,22].

Colour change has been described for at least eight Brazilian native species, but in none of the cases was the underlying mechanism characterised. The well-determined stages of flower colouring pattern in *P. raddianum* raise interesting questions about the genetic and chemical mechanisms involved in the regulation of this phenomenon. Here, to address this issue, *P. raddianum* flowers were analysed by profiling: (1) mRNA levels of key enzyme encoding genes of the flavonoid biosynthetic pathway, (2) pigments and co-pigments, (3) metals and, (4) primary metabolites during development. The results showed that colour change in *P. raddianum* is mainly determined by anthocyanin accumulation and influenced by the presence of co-pigments. Additionally, alterations in metal content and primary metabolites bring cues about the colour modulation.

## 2. Results

### 2.1. Chemical Profile

**Figure 2:**
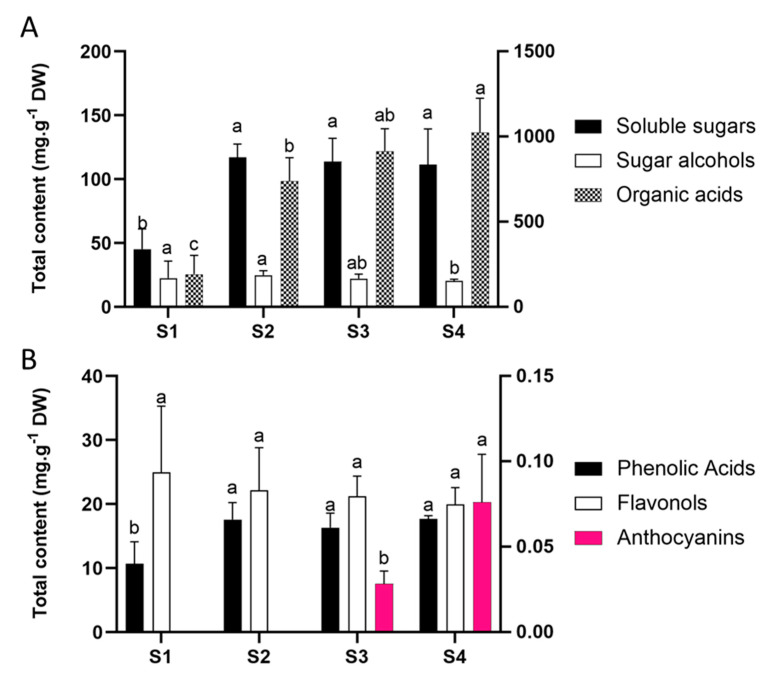
(**A**) Primary metabolites and (**B**) secondary metabolites during *Pleroma raddianum* flower development. The right scale represents soluble sugar content (**A**) and anthocyanins content (**B**). For each compound class, different letters indicate statistically significant differences (*p* < 0.05) between stages. Bars indicate standard deviation. S1—buds (day 0), S2—white flowers (day 1), S3—pink (day 2), S4—purple (day 3), DW—dry weight.

Primary metabolism comprises essential reactions for plant survival that involve the production and usage of a range of molecules including nucleic acids, amino acids, carbohydrates, and fatty acids. In particular, carbohydrate content directly affects the anthocyanin accumulation once they produce the building blocks for secondary metabolism [23]. Moreover, carbohydrates can act as signalling molecules in the regulation of transcription factors and structural genes of the anthocyanin pathway [24]. Sugars showed a three-fold increment from bud (S1) to white flower (S2) stage, maintaining high levels in pink stages (S3 and S4). Organic acids constantly increased from the S1 to S4 stage. In contrast, sugar alcohols were much less abundant and showed constant levels from S1 to S3, undergoing a slight reduction towards senescence (S4) (Figure 2A and Table S1).

In our previous work we performed a chemical screening of secondary metabolites in *P. raddianum* flowers identifying a diverse mixture of six phenolic acids, twenty flavonols, and two anthocyanins [2]. Here, we further quantified these phenolic constituents by UPLC-MS. Flavonols were the most abundant pigments ranging from 20 to 25 mg g^−1^ dry weight (DW), followed by phenolic acids that ranged from 10 to 18 mg g^−1^ DW, and anthocyanins from 0.028 to 0.076 mg g^−1^ DW (Figure 2B and Table S2).

Pigment abundance during flower development revealed that the amount of total phenolic acids increases during the transition from S1 to S2, maintaining constant levels from S2 onwards. Flavonols showed invariable levels and anthocyanins only appear in the pink stage, increasing from S3 to S4 (Figure 2B).

**Figure 3:**
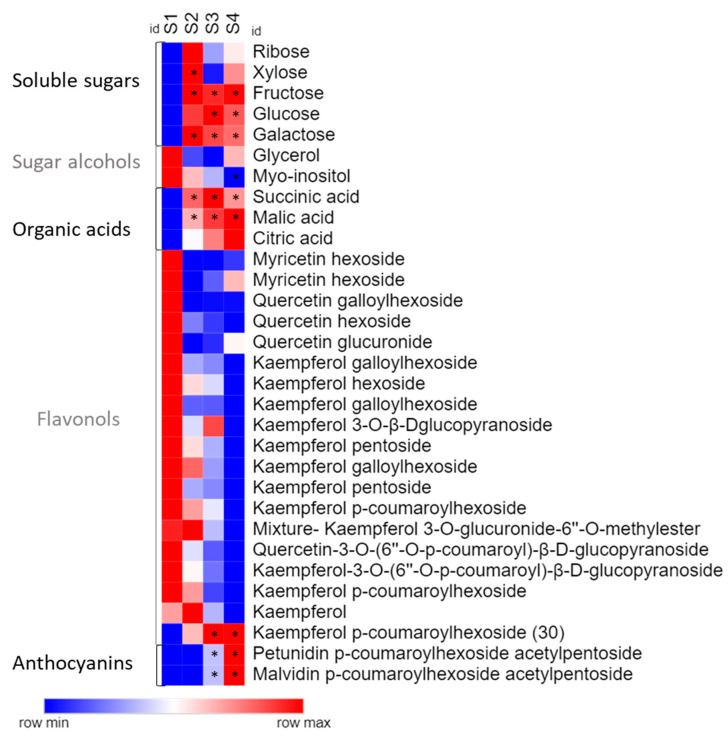
Heat map representing metabolic profile during the flower colour change in *Pleroma raddianum*. For each compound, asterisks indicate statistically significant differences compared to S1 stage. Data of sugars and organic acid were obtained by chromatography coupled to mass spectrometry (GC-EIMS) and flavonoid data by Ultra Performance Liquid Chromatography-High-Resolution Mass Spectrometry (UPLC-HRMS). S1—buds (day 0), S2—white flowers (day 1), S3—pink (day 2), S4—purple (day 3).

Analysing the compounds individually, soluble carbohydrates such as fructose, glucose, and galactose increased their levels from opened white flower (S2) onward, while *myo*-inositol was the only sugar that was reduced during flower development (Figure 3 and Table S1). Organic acids, such as malic and succinic acid, showed a significant increase from the bud to the pink flower stage.

All flavonols showed a decreasing trend, except the kaempferol *p*-coumaroylhexoside (30) that increased in stages S3 and S4. Both identified anthocyanins were only detected in the S3 stage, exhibiting a significant increment in S4 (Figure 3 and Table S2).

### 2.2. Gene Cloning and Expression Analysis

Having demonstrated that there is a differential accumulation of anthocyanins during flower development, we further investigated whether this observation could be explained by the differential mRNA profile of flavonoid biosynthetic genes. Since no genomic sequences are available for *P. raddianum*, we first cloned partial sequences for some key enzymes encoding genes that have been reported to be transcriptional regulated in other species [25,26]: *PHENYLALANINE AMMONIUM LYASE* (*PAL*), *CINNAMATE 4-HYDROXYLASE* (*C4H*), *CHS*, *FLAVONOL SYNTHASE* (*FLS*), and *ANTHOCYANIDIN SYNTHASE* (*ANS*). Additionally, two constitutive genes previously used for expression analyses in *Petunia hybrida* [27] were also selected to be used as reference genes in RT-qPCR experiments. The cloning strategy was based on the identification of conserved protein domains and the design of degenerated primers based on the *Eucalyptus grandis* W. Hill (Myrtaceae) sequences, the *P. raddianum* closest species with a completely sequenced genome. Gene fragments with approximately the predicted size were amplified (Figure S1 and Table S3), cloned, and sequenced.

The identity of the obtained gene fragments was corroborated by a phenetic analysis. The topology did not strictly follow the species phylogenetic relationship. However, the sequences from species belonging to the same order grouped together: *Brassica rapa* L. and *Arabidopsis thaliana* L. (Brassicales), *Medicago trunculata* Godr. and Gren. and *Trifolium pratense* L. (Fabales), and *P. raddianum* and *E. grandis* (Myrtales) (Figure S2), demonstrating that the strategy was successful to clone partial sequences of the genes of interest.

**Figure 4:**
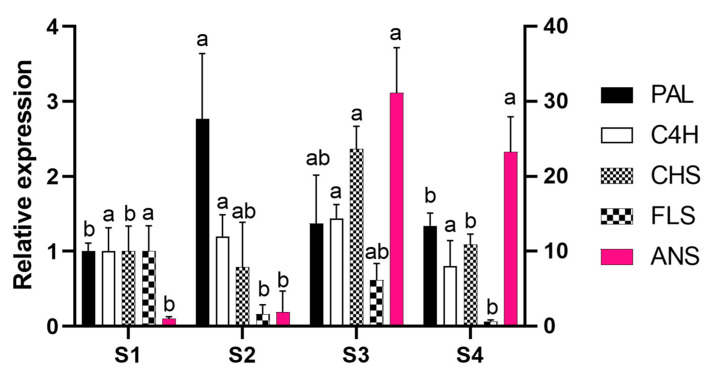
Expression profile of *PHENYLALANINE AMMONIUM LYASE* (*PAL*), *CINAMMATE 4-HYDROXYLASE* (*C4H*), *CHALCONE SYNTHASE* (*CHS*), *FLAVONOL SYNTHASE* (*FLS*), and *ANTHOCYANIDIN SYNTHASE* (*ANS*) genes during flower development. Data were normalised to expression of *ELONGATION FACTOR 1α* (*EF1*) and *RIBOSOMAL PROTEIN S13* (*RPS*) and are expressed related to S1 stage. The right scale characterises ANS expression. Bars indicate standard deviation. For each gene, different letters indicate statistically different relative expression ratios. S1—buds (day 0), S2—white flowers (day 1), S3—pink (day 2), S4—purple (day 3).

To address whether the transcriptional regulation of gene expression is involved in *P. raddianum* flower colour change, the mRNA accumulation of the cloned enzyme-encoding genes was profiled in the four flower stages analysed (Figure 4). As expected, since the gene fragments were cloned from petal cDNA, the presence of mRNA from all five genes was identified. Except for *C4H* that maintained constant levels of mRNA, the other four genes analysed were shown to be transcriptionally regulated during flower development.

The expression of the first committed gene in the phenolic compound biosynthetic pathway, *PAL*, peaked in S2, while *CHS* transcripts reached the highest level at S3. *FLS*, whose product diverges the route towards flavonol synthesis, showed maximum expression level in S1, decreasing at S2 and S4. Finally, the *ANS* enzyme encoding gene, responsible for the synthesis of the anthocyanin chromophores, is 30-fold upregulated from S2 to S3 and remains highly expressed until S4.

### 2.3. Metal Content

Due to the eventual impact of metal concentration on pigment colour, the metal content was also profiled during flower development. Among the analysed metals, Fe^3+^ and K^+^ were the most abundant micro and macronutrients, respectively (Table 1). The only metal that showed fluctuation between flower stages was Fe^3+^ ion, which showed a 3,5-fold increase from S1 to S4.

### 2.4. Colour Characterization of Petals

In order to gain further evidence about colour change, the colour parameters (CIELab) of *P. raddianum* flowers were evaluated in the opened flower stages. The colour profile was decomposed in terms of colour contributions: green (−a *)-red (+a *), blue (−b *)-yellow (+b *), lightness (L *), chromatic tonality (hue angle, *h_ab_*) and colour saturation (metric chroma, *C* *) (Table 2). Except for *h_ab_* that only differs between the white and pink stage, all other parameters showed that the stages are differently coloured. The red and blue colour (*a* * and *b* *) increased from S2 to S4, the same was observed with *C* *. Lightness peaked in S2, and drastically reduces with the colour appearance, and increases in S4.

## 3. Discussion

In this work, the phenomenon of *P. raddianum* floral colour change was investigated by a comprehensive analysis of pigments, gene expression, primary metabolites, and metal profiling during flower development. The flower colour is commonly related to the anthocyanin content in model plants such as petunia (*P. hybrida*) [28]. Moreover, anthocyanins accumulation was shown to be responsible for the coloured stages in the species whose corolla colour changes [4,5,6,12,13,14]. The results obtained here demonstrate that this is the case in *P. raddianum*, where petunidin and malvidin appear at the S3 stage together with the pink colour and are increased at the purple stage.

**Figure 5:**
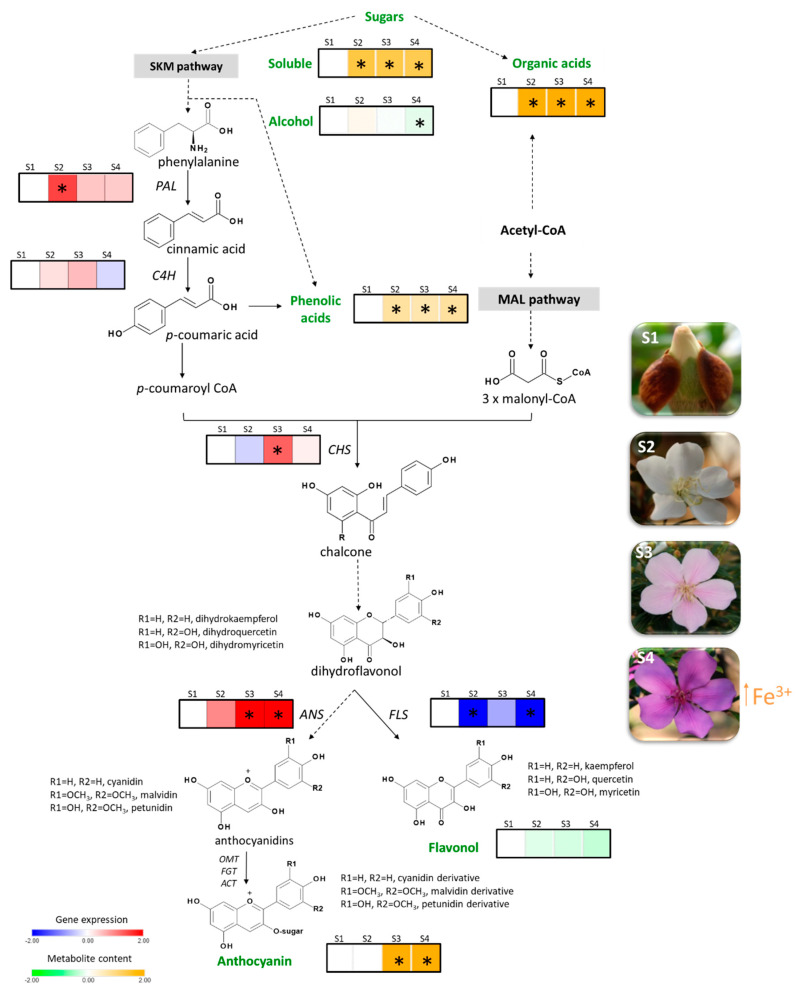
Comprehensive schematic representation of the obtained results. Flavonoid biosynthetic pathway showing heatmaps representing metabolites content (orange to green) and mRNA expression profile (blue to red) relative to the S1 stage. Dotted lines represent more than one enzymatic reaction. Quantified compounds are highlighted in green. Asterisks indicate statistically significant different values compare to the S1 stage. Abbreviations: *PHENYLALANINE AMMONIUM LYASE* (*PAL)*, *CINAMMATE 4-HYDROXYLASE (C4H)*, *CHALCONE SYNTHASE* (*CHS)*, *FLAVONOL SYNTHASE* (*FLS)*, *ANTHOCYANIDIN SYNTHASE (ANS*), *O-METHYLTRANSFERASE* (*OMT*), *FLAVONOID GLYCOSYL TRANSFERASE* (*FGT*) and *ANTHOCYANIDIN ACYL TRANSFERASE* (*ACT*) genes, S1—buds (day 0), S2—white flowers (day 1), S3—pink (day 2), S4—purple (day 3).

The genes that encode the enzymes involved in flavonoid biosynthetic pathway can be grouped into two classes: early biosynthetic genes (e.g., *PAL*, *C4H*, *CHS*, and *FLS*) and late biosynthetic genes (e.g., *DIHYDROFLAVONOL 4-REDUCTASE* (*DFR*) and *ANS*) [29]. Numerous studies have demonstrated the transcriptional regulation of flavonoid biosynthesis in plants, several transcription factors have even been identified. For example, the MBW transcription complex, composed of factors that belong to the MYELOBLASTOSIS (MYB), BASIC HELIX-LOOP-HELIX (bHLH), and BETA-TRANSDUCIN REPEAT (WD40) family of proteins were shown to be a master regulator of structural gene expression [30,31,32,33]. Gene expression analysis in *P. raddianum* flowers was a challenging enterprise because not only is the genome not sequenced, but there is no genomic sequence available for phylogenetically related species, with the most closely related species, *E. grandis*, being from a different family, Myrtaceae. Thus, the target genes were carefully chosen based on the secondary metabolites profile (Figure 2) and foundational publications that identified which genes are transcriptionally regulated [25,26]. The homology-based cloning approach used was successful and allowed the transcriptional profiling of four early (*PAL*, *C4H*, *CHS*, and *FLS*) and one late (*ANS*) gene of flavonoid biosynthesis that showed clear correlations with the pigment abundance pattern (Figure 5). *PAL* expression increases in the S2 stage, providing precursors for the increment in phenolic acids at this stage and the further accumulation of anthocyanins at S3 and S4. Interestingly, *FLS* was the only enzyme-encoding gene that showed a reduction in the expression profile from S1 onwards by following the decreasing trend observed for flavonols content, which were the most abundant pigments identified during the whole flower development. The first committed step towards the production of pink pigments is catalysed by the product of *ANS* gene. Its mRNA profile exactly coincided with that observed for anthocyanins, displaying an increment from S2 to S3 and maintaining high levels at the S4 stage and thus explaining the switch from white to pink. *CHS* also peaked at S3 boosting the production of anthocyanin precursors. The obtained results are in clear agreement with those reported for *V. cornuta*, where the expression of *CHS* and *DFR* increased during flower development, and the *ANS* is strongly upregulated at the final coloured stages [13]. Furthermore, our data are also in line with that observed in *N. mutabilis* flowers where *CHS* expression correlated with malvidin accumulation and the concomitant colour change from white to red [4]. Finally, an RNA-seq-based transcriptomic analysis aimed to understand the mechanism underneath the flower colour change from white to pink in *Paeonia ostii* demonstrated a positive correlation between the anthocyanin concentrations and the expression of *CHALCONE ISOMERASE* (*CHI*), *FLAVANONE 3-HYDROXYLASE* (*F3H*), *FLAVONOID 3′-HYDROXYLASE* (*F3′H*), *DFR,* and *ANS* biosynthetic genes, which exhibited higher levels of mRNA in pink than in white flower stages [5].

The regulation of flavonoid biosynthesis, in vegetative tissues, is intimately associated with environmental changes to enhance plant survival under stressful conditions, such as UV radiation, visible light, cold, osmotic stress, pollution, and pathogen infection. In particular, the role of the phytochrome-mediated signal transduction pathway, associated with temperature and light perception, has been abundantly explored [34,35,36,37]. In this sense, various phytochrome signalling components in *A. thaliana*, such as the PHYTOCROME INTERACTING FACTORS (PIFs) and PROTEIN LONG HYPOCOTYL 5 (HY5), have been shown to act as regulators of anthocyanin biosynthesis [38,39,40,41]. In other plants, this relationship between light perception and anthocyanin accumulation has also been addressed [42,43,44]. In *P. raddianum,* a preliminary experiment showed that when the plants were maintained indoor under low light irradiance, the flowers did not homogenously turn from white to pink, and fell in 24 h (Figure S3), suggesting a regulatory role of light in flower anthocyanin accumulation in this species. Reinforcing our observation, in cotton flowers (*Gossypium* sp., Malvaceae), which turn from white to pink, gene expression analysis has shown that light and shade regulate anthocyanin biosynthesis, in particular the *ANS* gene [45]. Likewise, *Viola cornuta* flowers did not undergo colour change in darkness [13].

Anthocyanin co-pigmentation is a natural phenomenon that enhances the stability of the flavylium cation from nucleophile attack, preventing anthocyanin degradation. For example, metalloanthocyanin, which is associated with a blue colour pattern, is a supramolecular and self-assembled metal complex pigment, composed of anthocyanins, flavones, and metals [19,46]. Interestingly, the only flavonol whose accumulation profile accompanied that observed for both identified anthocyanins was kaempferol *p*-coumaroylhexoside, exhibiting significant increment from S2 to S3, suggesting that this flavonol may act as a co-pigment in pink flowers, while petunidin and malvidin content drives the colour in *P. raddianum* (Figure 3 and Figure 5). Additionally, we have identified an increment in Fe^3+^ concentration accompanying the transition from S3 to S4 (Figure 5 and Table 1) flower stages. Even though *P. raddianum* has no visible blue colour, colorimetric parameters determining the blue tonality (−*b* *) increased from S3 to S4 (Table 2), as well as lightness and saturation of colour. Although co-pigmentation and metal complexation in this species remain to be further experimentally proven, our data allowed us to propose the formation of flavonoid–Fe^3+^ complexing from S3 to S4, stabilising the pigments. Furthermore, in *P. raddianum*, both anthocyanins are diacyl and diglycosidic compounds, which seem to favour chelates [47]. Sigurdson et al. [47] investigated colour expression and the stability of Al^3+^ and Fe^3+^ chelates in pH 6–7 by spectrophotometry (380–700 nm) and colorimetry (CIELab). Larger bathochromic shifts (changes in UV-Vis absorption spectra to higher wavelengths) were associated to these two metals for all cyanidin derivatives tested in pH 6, indicating that a slight acid pH change enhances metal chelation. Moreover, cyanidin aliphatic acylated derivatives exhibited the smallest spectral shift when compared to aromatic acylated derivatives, indicating that aromatic acylation and glycosylation of the anthocyanin are more propitious to metal chelation.

Vacuolar pH measurement is extremely difficult because it is necessary a proton-selective microelectrode [20,48]; otherwise, the common cell homogenised or isolated protoplast pH analysis does not precisely reflect vacuolar pH [46]. Although pH was not directly measured, our results provided evidence about vacuole acidification in *P. raddianum.* A dramatic increment of succinic and mainly malic acids from S1 towards S3 (Figure 3 and Figure 5) strongly suggests that pH reduction might contribute to flower colour in this species. Similarly, in *Rhododendron schlippenbachii* Maxim. (Ericaceae) and *Nelumbo nucifera* Gaertn. (Nelumbonaceae) flowers, by employing a metabolomic approach, authors concluded that coloured flowers are directly related to the accumulation of organic acids, such as succinic and malic acid [49,50].

The metabolomic approach allowed the detection of carbohydrates, whose derivatives are the building blocks and energy source for flavonoid biosynthesis (Figure 5). Usually sugar content in flowers reduces them from white to coloured stages, reflecting the use of energy and carbon to promote anthocyanin production and glycosylation [49,50]. Contrarily, in *P. raddianum* an increment in sugar content was verified in the S2 stage that remained high until S4. It is important to highlight that no senescent stages of flower development were investigated in our study and, thus, these carbohydrates might be still feeding flavonoid biosynthesis.

In this work, by applying a combined approach of detailed biochemical and molecular genetic techniques, we characterised the pigment profile and investigated the regulation of colour change in *P. raddianum* flowers. Phenolic compounds were quantified showing variations in pigment and co-pigment contents during flower development. The partial cloning of five key genes of the flavonoid biosynthetic pathway allowed us to profile mRNA levels that explained, at least in part, the pigment accumulation pattern. Together these data suggest that the flower colour change in *P. raddianum* is regulated by the transcriptional control of the structural genes of the flavonoid biosynthetic pathway, mainly *CHS* and *ANS*, that results in the accumulation of petunidin and malvidin, as well as the co-pigment kaempferol *p*-coumaroylhexoside. Moreover, the quantification of metal contents suggested that Fe^3+^ might influence the saturation of the colour at the purple stage. Finally, organic acids, in particular malic acid, are probably involved in vacuole acidification, which might promote a better environment for metal chelation.

Several analyses could complement these findings about the mechanism of colour change in *P. raddianum*. The identification of the transcriptional factors that control flavonol/anthocyanin accumulation would shed light on the differential expression pattern identified for the analysed genes. Additionally, genetic analysis of the late reactions, such as methylations, glycosylations and acylations of flavonoid structures, would bring information about the substituents identified. It is worth mentioning that the ubiquity of these reactions renders the identification of the specific enzyme encoding genes (*ANTHOCYANIDIN ACYL TRANSFERASE*, *FLAVONOID GLYCOSYL TRANSFERASE*, *O-METHYLTRANSFERASE*) extremely difficult. Due to the demonstrated post-transcriptional regulation of some flavonoid biosynthetic genes [51,52,53,54,55,56], the study of the enzymatic activity would increase the knowledge about this phenomenon. Finally, the effect of Fe^3+^ and vacuolar pH on flower colour needs to be better understood by means of addressing the subcellular localisation and the formation of metal–flavonoid complexes.

In conclusion, the generated information contributed to the understanding of the flower colour change phenomenon in a Brazilian native species and constitute repository data for future studies with Melastomataceae.

## 4. Materials and Methods

### 4.1. Plant Material

Petals at the S1 to S4 stages (Figure 1) were sampled from five *Pleroma raddianum* (cv. “manacá-anão”) plants located in Praça Carlos José Gíglio (Latitude: −23.57998, Longitude: −46.73403) in the most vigorous flowering period (May and June/2016) between 8 and 9 a.m. For each developmental stage, a pool of petals (~5 g) from each plant was considered a biological replicate (n = 5). Samples were collected, immediately frozen in liquid nitrogen, ground, and stored at −80 °C for mRNA extraction or freeze dried for chemical analysis. For gene cloning, a pool of all stages collected in May and June/2014 was used. An exsiccate was deposited in the herbarium of Institute of Bioscience (SPF) of the University of São Paulo (ID: Furlan73).

### 4.2. Metabolic Profile

#### 4.2.1. Pigment Profile

Phenolic compounds were extracted from 100 mg of petal powder with 1.5 mL of 0.2 % HCl in methanol (MeOH). The samples were sonicated for 10 min and centrifuged at 10,000 rpm for 10 min. The supernatant was collected and the pellet extracted twice. The extract was filtered (PTFE 0.45 μm) and analysed by a Ultra Performance Liquid Chromatography (UPLC) system with a Diode Array Detector (DAD) (Dionex Ultimate 3000) and an Electrospray Ionisation Quadrupole Time-of-Flight High-Resolution Mass Spectrometry (ESI-QTOF-HRMS) detector (Bruker Maxis, Bremen, Germany), MS/MS analysis was performed with a Broadband Collision Induced Dissociation (bbCID) detector (Bruker, Bremen, Germany). Separation was achieved by using a C18 column (Waters Acquity UPLC 100 × 2.1 mm^−1.7^ µm) at a flow rate of 0.3 mL min^−1^, and 4 µL of injection volume, the column temperature was 45 °C, and the solvent system composed of 1% formic acid in water (A) and 1% formic acid in acetonitrile (B). Gradient elution: 5 to 25% of B (0–40 min), 25 to 100% of B (40–42 min), 100% of B (42–42.5 min), 100 to 5% of B (42.5–43 min), 5% of B (43–46 min). Separated compounds were first monitored using DAD (200 to 600 nm) and then MS scans were performed in positive ion mode (MS+), in the range *m/z* 75–1250 *m/z*, in the following conditions: capillary voltage set to 4500 V, end plate offset at −500 V, nebuliser at 2 Bar, dry gas 12 L min^−1^ and dry gas temperature at 200 °C MS was calibrated using sodium formate. All data were processed using Data analysis software 4.2 (Bruker, Bremen, Germany). To quantify the compounds, the areas of MS^+^ chromatograms were normalised and compared with standard curves of *p*-coumaric acid, kaempferol and cyanidin (Table S4). To identification details see Rezende et al. [2].

#### 4.2.2. Primary Metabolism Profile

Petal material (20 mg) was extracted in 500 µL of methanol/chloroform/water (12:5:1, v/v) and ribitol 0.2 mg mL^−1^ (50 µL) was added as internal standard (modified from Suguiyama et al. [57]). The polar phase was derivatised using methoxyamine hydrochloride (28 μL) for 2 h at 37 °C and N-Methyl-N-(trimethylsilyl) trifluoroacetamide (48 μL, MSTFA) for 30 min at 37 °C. Samples were analysed using gas chromatography coupled to mass spectrometry (GC-EIMS Agilent Technologies, Santa Clara, CA, USA) with a capillary column VF-5MS column (Agilent, Santa Clara, CA, USA, length 30 m × 250 μm × 0.25 μm) and a pre-column (0.25 mm × 10 m). The injection volume was 1 μL using Helium as mobile phase (1.0 mL min^−1^). Temperature was programmed as isothermal for 5 min at 70 °C, followed by a 5 °C per min ramp to 295 °C. The injector, ion source, and quadrupole temperatures were 230 °C, 200 °C, and 150 °C, respectively. The EIMS analysis employed an ionisation voltage of 70 eV and mass range recorded was of *m/z* 50 to *m/z* 600 at 2 scan/s. Substances were identified and compared with authentic standards using National Institute of Standards and Technology (NIST, v2.0, 2008) and Global Natural Products Social Molecular Networking (GNPS, 2016) spectral libraries. To identify the compounds, the Linear Index of Retention was calculated using the alkane standard according to Viegas and Bassoli [58] and compared with the Golm Metabolome Database (GMD) and PubChem. To quantify the compounds, the areas of MS chromatograms were compared with internal standard.

### 4.3. Gene Cloning and Expression Profile

#### 4.3.1. Sequence Analysis for Primers Design

The amino acid sequences encoded by the functionally characterised genes *PAL* (AT2G37040.1), *C4H* (AT2G30490.1), *CHS* (AT5G13930.1), *FLS* (AT5G08640.1) and *ANS* (AT4G22880.1) from *Arabidopsis thaliana* [59,60,61,62] were used as query to identify, by the tBLASTx program [63], the orthologous sequences from *Vitis vinifera*, *Eucalyptus grandis*, and *Solanum lycopersicum*, fully sequenced genomes available in the Phytozome v 9.0 database [64]. The identified amino acid sequences were aligned using the MUSCLE program following the standard configurations of the MEGA 6 package [65]. After the identification of conserved domains, the alignments were converted to nucleotides for degenerated primer design based on the corresponding *Eucalyptus grandis* sequences, which is the closest related species to *P. raddianum* with a completely sequenced genome (Figure S4 and Table S5). The quality of the designed primers was verified using the software Oligo Analyser 3.1 [66].

By using the same strategy described above, primers were designed for the cloning of two reference genes for Reverse Transcriptase Quantitative Polymerase Chain Reaction (RT-qPCR) normalisation. The chosen genes were those previously described for gene expression analyses in *Petunia hybrida* flowers, *ELONGATION FACTOR 1α* (*EF1*) and *RIBOSOMAL PROTEIN S13* (*RPS*) [27].

#### 4.3.2. RNA Extraction and cDNA Synthesis

Total RNA from 3 g of petal samples was extracted with Cetyl trimethylammonium bromide (CTAB) [67] (Figure S5 and Table S6). DNA was removed with 100 U of amplification-grade DNase (Invitrogen) following the recommended protocol. cDNA was synthesised from 1 µg of RNA using oligo dT (for gene cloning) or random primers (for RT-qPCR) and the SuperScript III kit (Invitrogen). cDNA quality was confirmed by PCR using *RPS* primers.

#### 4.3.3. Cloning

Gene fragments were amplified by PCR in a total volume of 50 μL containing 0.2 mM each dNTPs, 0.2 µM of each primer (Table S5), 1X reaction buffer, 1.5 mM MgCl_2_, 50 ng of cDNA, and 2 U of Taq DNA polymerase (Invitrogen). The amplification conditions were: 94 °C for 3 min, 35 cycles of 94 °C for 30 s, 50 °C for 30 s, and 72 °C for 1 min, and then 72 °C for 10 min. Amplification products were purified (Kit GFX Amersham Biosciences, Buckinghamshire, United Kingdom) and cloned into a TOPO-TA vector (Dual promoter Kit Invitrogen) following the recommended protocols. Transformation was carried by using 50 μL of DH10B strain competent cells with 2 μL of the ligation product. The mixture was kept on ice for 30 min and subjected to a thermal shock of 45 sec at 65 °C and 2 min on ice. An amount of 500 μL of Super Optimal Broth (SOC) medium [68] was added for a shaking incubation (45 min at 37 °C). Samples were centrifuged (3 min, 3000 rpm at room temperature) and cells resuspended and plated on Luria-Bertani (LB) agar [69] with kanamycin, 5-bromo-4-chloro-3-indoxyl-β-D-galactopyranoside (X-Gal) and isopropil β-D-1-tiogalactopiranosida (IPTG). Five white colonies for each fragment were grown in liquid LB and, plastidial DNA was purified and sequenced with vector universal primers.

#### 4.3.4. Sequence Analyses

To verify the identity of the cloned *P. raddianum* gene fragments, phenetic analyses were performed. Orthologous sequences from *Arabidopsis thaliana* (AT) and *Bra rapa* L. (Brara)—Brassicaceae, *Eucalyptus grandis* (Eucgr)—Myrtaceae, *Mendicago trunculata* (Medtr) and *Trifolium pratense* L. (Tp)–Fabaceae, and *Solanum lycopersicum* (Soly)—Solanaceae, were retrieved from the Phytozome v 12.1 database [70] by the tBLASTx program [63]. The species were selected according to the following criteria: (i) fully sequenced genome, (ii) genes belonging to Phytozome gene families whose functional annotation was associated to phenolic compound biosynthesis (gene family 94476114 for *PAL*, 94469571 for *C4H*, 94475332 for *CHS*, 94475392 for *FLS*, 94470119 for *ANS*, 94475526 for *RPS* and 94476231 for *EF1*), and (iii) species placed in Rosids clade in Angiosperm Phylogeny Group (APG) IV classification (AT, Brara, Eucgr, Medtr and Tp, and Soly as the outgroup). The coding sequences were aligned using the Clustal program following the standard configurations of the MEGA 6 package [65], with manual verification of codon alignment according to the amino acid sequence. The phenograms were constructed with the following parameters: *Neighbor-joining*, *Bootstrap* of 1000 replicates and the best model test for each analysis. Gene sequences were submitted to gene bank under the following numbers: MW000889 for PAL, MW000890 for C4H, MW000891 for CHS, MW000892 for FLS, MW000893 for ANS, MW000894 for EF1 and MW000895 for RPS.

#### 4.3.5. RT-qPCR

Based on *P. raddianum* sequences, gene specific primers for expression analysis were designed to amplify fragments of approximately 160 bp (Table S5). To quantify mRNA levels by RT-qPCR, the reactions were carried out in a QuantStudio 6 Flex Real-Time PCR system (Applied Biosystems) using 2X Power SYBR Green Master Mix reagent (Life Technologies) in 14 µL final volume. Absolute fluorescence data were analysed using the LinRegPCR software package [71] to obtain quantitation cycle (Cq) values and calculate primer efficiencies (the primer efficiency values ranging from 1.85 to 1.98). Relative expression values were obtained normalising against the geometric mean of the two reference genes (*EF1* and *RPS*), according to the formula below [72]:

(1) RE=Pegoi^ (control Ct mean −sample Ct mean)Peref^ (control Ct mean −sample Ct mean)

where RE is relative expression, Pe is primer efficiency, goi is gene of interest, Ct is treshold cycle, control Ct is the Ct mean of the biological control, sample Ct is the Ct mean of biological sample and ref are the reference genes.

A permutation test lacking sample distribution assumption [73] was applied to detect statistical differences (*p* < 0.05) in expression ratios using fgStatistics software package [74].

### 4.4. Metal Content

To analyse the content of metals, 500 mg of freeze-dried petal powder were digested with nitro-perchloric and the contents of Mg^2+^, K^+^, Na^+^, Ca^2+^, Cu^2+^, Mn^2+^, Zn^2+^ and Fe^3+^ were determined by atomic absorption spectroscopy in the Laboratory of Vegetal Tissue (Luiz de Queiroz College of Agriculture, University of Sao Paulo—ESALQ, USP).

### 4.5. Flower Colour

Five fresh petals, from stages S2, S3 and S4, were measured three times at adaxial surface. Petal colour was assessed by using a Konica Minolta CR-400 colorimeter, to measure lightness (L *), and two chromatic components a * and b * of the CIE*Lab* colour scale. The measurements were performed indoors in a daylight condition. Hue angle (H_ab_) and metric chroma (C *), were calculated according to the following equations [75]:

C * = (a *^2^ + b *^2^)^1/2^ (2)

H_ab_ = tang^−^^1^(b */a *) (3)

### 4.6. Data Analyses

Statistical analysis for pigment and metal profiles was performed using Infostat package [74]. When the data set showed normality, ANOVA followed by the Tukey test (*p* < 0.05) were performed to compare differences between groups. In the absence of normality, a non-parametric comparison was performed by applying the Kruskal-Wallis test (*p* < 0.05). All values represent the mean of five biological replicates.

## Figures and Tables

**Table 1:** Metal quantification during *Pleroma raddianum* flower development. S1—buds (day 0), S2—white flowers (day 1), S3—pink (day 2), S4—purple (day 3).

**Micronutrients**	**S1** (**mg g^−1^ DW**)	**S2** (**mg g^−1^ DW**)	**S3** (**mg g^−1^ DW**)	**S4** (**mg g^−1^ DW**)
Cu^2+^	0.006 ± 0.001 ^a^	0.007 ± 0.002 ^a^	0.006 ± 0.001 ^a^	0.008 ± 0.002 ^a^
Fe^3+^	0.062 ± 0.026 ^b^	0.116 ± 0.040 ^b^	0.114 ± 0.024 ^b^	0.220 ± 0.043 ^a^
Mn^2+^	0.012 ± 0.007 ^a^	0.010 ± 0.006 ^a^	0.007 ± 0.002 ^a^	0.010 ± 0.004 ^a^
Zn	0.019 ± 0.003 ^a^	0.025 ± 0.011 ^a^	0.021 ± 0.003 ^a^	0.025 ± 0.004 ^a^
**Macronutrients**	**S1** (**mg g^−1^ DW**)	**S2** (**mg g^−1^ DW**)	**S3** (**mg g^−1^ DW**)	**S4** (**mg g^−1^ DW**)
K^+^	10.207 ± 1.773 ^a^	11.245 ± 2.546 ^a^	9.731 ± 0.432 ^a^	11.029 ± 1.088 ^a^
Ca^2+^	2.357 ± 0.402 ^a^	2.251 ± 0.5111 ^a^	2.099 ± 0.265 ^a^	2.051 ± 0.447 ^a^
Mg^2+^	1.169 ± 0.276 ^a^	1.181 ± 0.289 ^a^	0.980 ± 0.131 ^a^	0.9028 ± 0.083 ^a^
Na^+^	0.969 ± 0.378 ^a^	0.829 ± 0.244 ^a^	1.020 ± 0.208 ^a^	0.956 ± 0.483 ^a^

Data were acquired by Atomic Absorption Spectroscopy. Elements highlighted in bold showed statistically significant differences between stages. Letters indicate statistically significant different values in each line. DW—dry weight.

**Table 2:** Flower colour parameters in *Pleroma raddianum* petals. S2—white flowers (day 1), S3—pink (day 2), S4—purple (day 3).

	S2	S3	S4
*a* *	−1.93 ± 0.60 ^c^	21.31 ± 1.31 ^b^	41.59 ± 3.97 ^a^
*b* *	7.544 ± 0.88 ^c^	−10.69 ± 0.58 ^b^	−20.47 ± 1.04 ^a^
*L* *	93.92 ± 0.28 ^a^	46.36 ± 3.99 ^c^	64.65 ± 2.32 ^b^
*h_ab_*	104.09 ± 3.31 ^b^	333.35 ± 0.35 ^a^	333.72 ± 1.14 ^a^
*C* *	7.80 ± 0.98 ^c^	23.85 ± 1.42 ^b^	77.77 ± 0.68 ^a^

Petals’ colour were assessed by using a Konica Minolta CR-400 colorimeter, to measure colour contributions of the CIELab colour scale. Letters indicates abbreviation of colour contributions: a *: green (−a *)–red (+a *); b *: blue (−b *)–yellow (+b *); lightness (L *); chromatic tonality (hue angle, *h_ab_*); and colour saturation (metric chroma, *C* *).

## Supplementary Materials

The following are available online at, Figure S1. Agarose gel (0.8%) showing the cloned fragments. The abbreviations indicate: *PHENYLALANINE AMMONIUM LYASE* (*PAL*), *CINAMMATE 4-HYDROXYLASE* (*C4H*), *CHALCONE SYNTHASE* (*CHS*), *FLAVONOL SYNTHASE* (*FLS*) and *ANTHOCYANIDIN SYNTHASE* (*ANS*), *ELONGATION FACTOR 1*-α (*EF1*) and *RIBOSSOMOAL PROTEIN S13* (*RPS*) genes. Figure S2. Phenograms for PHENYLALANINE AMMONIUM LYASE (PAL) (A), CINAMMATE 4-HYDROXYLASE (C4H) (B), CHALCONE SYNTHASE (CHS) (C), FLAVONOL SYNTHASE (FLS) (D), and ANTHOCYANIDIN SYNTHASE (ANS) (E) amino acid sequences. Only sequences from species with full sequenced genomes were included in the analyses: *Eucalyptus grandis* (Eucgr), *Arabidopsis thaliana* (AT), *Brassica rapa* (Brara), *Medicago trunculata* (Medtr), *Trifolium pratense* (Tp) and *Solanum lycopersicum* (Soly). Trees were constructed using the following parameters: Neighbor-joining, Bootstrap of 1000 replicates and the best model test for each analysis. Grey boxes show the clusters for *Pleroma raddianum* (T.pulchra) and E. grandis sequences. Figure S3. *Pleroma raddianum* flowers need light to turn from white to purple colour. Plants were maintained indoors under low light irradiance. After 24 h (A to B) white flowers did not homogenously turn to pink (D) and fell down the following day (B to C). (E). Normal pink flower at the S3 stage. Figure S4. Alignments used for primer design to clone the partial gene sequences of *PHENYLALANINE AMMONIUM LYASE* (*PAL*) (A), *CINAMMATE 4-HYDROXYLASE* (*C4H*) (B), *CHALCONE SYNTHASE* (*CHS*) (C), *FLAVONOL SYNTHASE* (*FLS*) (D) and *ANTHOCYANIDIN SYNTHASE* (*ANS*) (E). Boxes indicate the primers sequences. Figure S5. RNA integrity. Agarose gel (1%) for analysis of RNA integrity, approximately 500 µg of each sample was loaded. The numbers indicate the biological replicates (1 to 5) for each stage (S1 to S4). S1—buds (day 0), S2—white flowers (day 1), S3—pink (day 2), S4—purple (day 3). Table S1. Sugar, organic acids and phenolic acids analysed by GC-EIMS in each developmental stage of *Pleroma raddianum* flowers. S1—buds (day 0), S2—white flowers (day 1), S3—pink (day 2), S4—purple (day 3). Table S2. Pigment profile during *Pleroma raddianum* flower development. S1—buds (day 0), S2—white flowers (day 1), S3—pink (day 2), S4—dark pink (day 3). Table S3. Identity of obtained cDNA fragments from *Pleroma raddianum* with *Eucalyptus grandis* sequences. Table S4. Standard curves parameters. Table S5. Primers used for gene cloning and RT-qPCR. Table S6. RNA quantification by nanodrop. The numbers indicate the biological replicates (1 to 5) for each stage (S1 to S4). S1—buds (day 0), S2—white flowers (day 1), S3—pink (day 2), S4—purple (day 3).
